# Use of rapid molecular TB diagnostics for incarcerated people in Brazil

**DOI:** 10.5588/ijtld.22.0642

**Published:** 2023-05-01

**Authors:** E. B. Fajer, F. D. Costa, D. M. Pelissari, F. A. Diaz Quijano, A. Coelho de Brito, E. A. T. Cunha, J. Croda, J. R. Andrews, K. S. Walter

**Affiliations:** 1 Stanford University School of Medicine, Stanford, CA, USA; 2 National Tuberculosis Control Program, Brasília, DF, Brazil; 3 Department of Epidemiology, School of Public Health, University of São Paulo, São Paulo, SP, Brazil; 4 Central Laboratory of Public Health, Campo Grande, MS, Brazil; 5 Federal University of Mato Grosso do Sul, Campo Grande, MS, Brazil; 6 Oswaldo Cruz Foundation, Campo Grande, MS, Brazil; Brazil; 7 Yale School of Public Health, New Haven, CT, USA; 8 University of Utah, Division of Epidemiology, Salt Lake City, UT, USA

Dear Editor,

In 2021, an estimated 4.2 million cases of TB remained undiagnosed.^1^ Reducing this gap through early diagnosis of disease and rapid detection of drug resistance is critical for managing individual outcomes and preventing onwards transmission of *Mycobacterium tuberculosis*.^1^–^3^ Expanding access to highly sensitive and specific rapid molecular TB diagnostics recommended by the WHO is an urgent priority for incarcerated individuals, who have a TB incidence 10 times that of the general population.^4^ In the Americas, TB notifications are increasingly concentrated within prisons,^5^,^6^ including in Brazil where 12.2% of the country’s notified TB cases occurred among incarcerated individuals in 2019.^7^ Although the National Tuberculosis Program in Brazil recently endorsed the use of Xpert^®^ MTB/RIF (MTB/RIF; Cepheid, Sunnyvale, CA, USA), rapid molecular diagnostics usage in prisons has not yet been measured.

We therefore quantified the use of Xpert and chest radiography (CXR) among all newly diagnosed TB cases reported to Brazil’s national notifiable disease system (*Sistema de Informação de Agravos de Notificação* or SINAN) from January 2015 to December 2018. We excluded cases with unknown incarceration status or patients below 18 years old, as they compose a small proportion of the incarcerated population. SINAN reports diagnostic results by testing modality, including MTB/RIF, CXR, sputum smear, and culture.^7^ Rapid molecular diagnostics are recommended by the Brazil Ministry of Health and the WHO, but, in practice, they are not uniformly available within prisons. MTB/RIF test outcomes were reported as *M. tuberculosis* detected, susceptible or resistant to rifampicin; *M. tuberculosis* not detected; inconclusive test; or not performed. Xpert G4 (Cepheid) was primarily used during our study period; Brazil subsequently introduced Xpert Ultra (Cepheid) across the country’s laboratory network in 2019. CXR outcomes were reported as suspect, normal, other pathology, or not performed; sputum smear outcomes as positive, negative, not performed, or not applicable. Culture outcomes were reported as positive, negative, in-progress, or not performed. For each diagnostic test, we classified individuals with positive, negative, or in-progress reported results as having received that test (test usage), and individuals with tests marked not performed or missing information as having not received that test (no test usage). We compared the use of different testing modalities between incarcerated and non-incarcerated populations with two-proportion *Z*-tests. We assessed heterogeneity in diagnostic usage between states with χ^2^ tests for equality of proportions. An ‘empty’ multilevel regression^8^ was run with state or municipality random effects and no other predictor variables to estimate intraclass correlation coefficients (ICCs) for municipality and state-level clustering, measuring the proportion of total variability as determined using cluster membership. We combined individual-level variables from SINAN with municipality-level covariates, including the 2010 average per capita household income and GINI coefficient, from Brazil’s Human Development Atlas,^9^ incarcerated population size from Brazil’s National Prison Information Survey,^10^ and total population density from the Brazilian Institute of Geography and Statistics.^11^ Finally, to identify locations with large diagnostic gaps, we calculated the proportions of incarcerated individuals diagnosed without MTB/RIF or CXR in the 26 municipalities with incarcerated populations over 5,000 individuals. This study was approved by the Institutional Review Board at Stanford University, Stanford, CA, USA (Protocol #50466).

**Figure 1:**
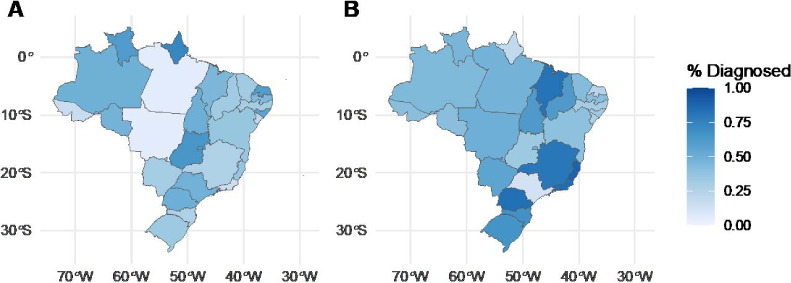
Spatial heterogeneity in the use of Xpert^®^ MTB/RIF and chest radiography among the incarcerated population. Map of Brazilian states showing the percentage of incarcerated individuals diagnosed with A) Xpert MTB/RIF; and B) chest radiography between 2015 and 2018. Darker colors represent greater diagnostic use.

**Figure 2:**
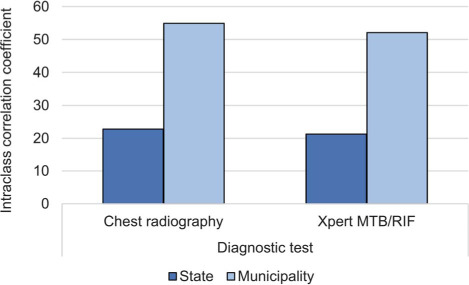
The variance in use of Xpert^®^ MTB/RIF and chest radiography attributed to state (dark grey; dark blue in online version) and municipality (light grey; light blue in online version) random effects as measured by the intraclass correlation coefficient.

Between 2015 and 2018, there were 284,415 newly diagnosed TB cases reported to SINAN, including 258,014 cases among patients over 18 years old with information on incarceration status. Of these, 10.6% (27,400/258,014 cases) occurred among incarcerated individuals and 89.4% (230,614/258,014) were among the general population. We found overall MTB/RIF use of 27.5% (70,988/258,014), CXR use of 70.9% (183,058/258,014), sputum smear use of 71.5% (184,509/258,014), and culture use of 34.1% (87,905/258,014), with significant overlap in populations receiving multiple different diagnostic tests. TB patients received a mean of 2.04 diagnostic tests. Use of MTB/RIF and CXR differed significantly between Brazil’s incarcerated and non-incarcerated populations. MTB/RIF use in incarcerated individuals was greater than that in non-incarcerated individuals (36.2% vs. 26.5%, *P* < 0.001) each year and overall, and its use in prison settings increased over the 4 years (*P* < 0.001) for a positive linear trend in MTB/RIF use. CXR use was consistently lower in incarcerated individuals than in non-incarcerated individuals (41.6% vs. 74.4%, *P* < 0.001) each year and overall. We found significant heterogeneity in usage in prisons of both MTB/RIF (χ^2^ 2717.3, degrees of freedom [df] = 26, *P* < 0.001) and CXR (χ^2^ 9039.7, df = 26, *P* < 0.001) across states (Figure 1). Diagnosis with MTB/RIF ranged from 4.7% and 4.9% in Pará and Mato Grosso, to 64.5% and 72.4% in Goiás and Amapá. Similarly, diagnosis with CXR ranged from 11.7% and 18.9% in São Paulo and Amapá, to 84.5% and 88.4% in Rio de Janeiro and Espírito Santo. In total, 21.3% and 52.1% of variation in MTB/RIF use, as measured by ICCs, could be attributed to a patient’s state and municipality respectively (Figure 2). Similarly, respectively 22.8% and 54.9% of variation in CXR use among incarcerated populations can be attributed to a patient’s state and municipality. In a binomial regression model, MTB/RIF use was greater in 2017 (odds ratio [OR] 1.55, 95% confidence interval [CI] 1.42–1.69) and 2018 (OR 2.23, 95% CI 2.03–2.44, *P* < 0.001) than in 2015. The size of the municipality incarcerated population was associated with greater use of MTB/RIF (OR 1.76, 95% CI 1.49–2.08, *P* < 0.001), although average per capita income, Gini coefficient, and population density were not significantly correlated with MTB/RIF use. Additionally, we found that the Gini coefficient and per capita income were positively associated with CXR use, whereas incarcerated population size was negatively associated with CXR use. Of the 26 Brazilian municipalities with incarcerated populations larger than 5,000 individuals, seven reported that less than the national average of 36.2% of incarcerated patients were diagnosed using MTB/RIF, and 18 reported that less than the national average of 41.6% of incarcerated patients were diagnosed using CXR.

A limitation of our study was that we investigated the use of different diagnostics for confirmed cases of TB, rather than their overall use, as this information was not available. Our study also focused on the years preceding the COVID-19 pandemic, so we were unable to assess the impact of this on TB screening.

A significant and increasing proportion of TB notifications in Brazil occur among incarcerated populations,^6^ creating a health and human rights crisis. Continued expansion of access to MTB/RIF, particularly in municipalities with large incarcerated populations, should be an urgent priority for TB control programs. Systematic monitoring of TB diagnosis and treatment will critically inform expansion of TB control programs and infrastructure. Expanding access to molecular diagnostics is a global priority and several countries (including Uzbekistan^12^ and El Salvador^13^) have rapidly increased access to the MTB/RIF assay. Ultimately, targeted expansion of rapid molecular diagnostics for incarcerated populations and regions with limited diagnostic usage should be an urgent priority.
